# Phenol-Soluble Modulins Contribute to Early Sepsis Dissemination Not Late Local USA300-Osteomyelitis Severity in Rabbits

**DOI:** 10.1371/journal.pone.0157133

**Published:** 2016-06-08

**Authors:** Benjamin Davido, Azzam Saleh-Mghir, Frédéric Laurent, Claire Danel, Florence Couzon, Laure Gatin, François Vandenesch, Jean-Philippe Rasigade, Anne-Claude Crémieux

**Affiliations:** 1 Département de Médecine Aigüe Spécialisée, Hôpital Universitaire Raymond-Poincaré, Assistance Publique–Hôpitaux de Paris, Garches, and EA 3647, Faculté de Médecine Paris–Île-de-France Ouest, Université Versailles–Saint-Quentin, Versailles, France; 2 CIRI, International Center for Infectiology Research, Inserm U1111-CNRS UMR5308, ENS Lyon–Université Lyon 1, Hospices Civils de Lyon, Lyon, France; 3 Département de Pathologie, UFR de Médecine Paris 7, site Bichat, Paris, France; National Research Laboratory of Defense Proteins, REPUBLIC OF KOREA

## Abstract

**Introduction:**

In bone and joint infections (BJIs), bacterial toxins are major virulence factors: Panton—Valentine leukocidin (PVL) expression leads to severe local damage, including bone distortion and abscesses, while *α*-hemolysin (Hla) production is associated with severe sepsis-related mortality. Recently, other toxins, namely phenol-soluble modulins (PSMs) expressed by community-associated methicillin-resistant *Staphylococcus aureus* (CA-MRSA) strain USA300 (LAC WT) were shown to have *ex vivo* intracellular cytotoxic activity after *S*. *aureus* invasion of osteoblasts, but their *in vivo* contribution in a relatively PVL-sensitive osteomyelitis model remains poorly elucidated.

**Materials and Methods:**

We compared the outcomes of experimental rabbit osteomyelitises induced with *pvl*^+^*hla*^+^*psms*^+^ LAC WT and its isogenic *Δpsm* derivatives (LAC *Δpsmα* and LAC *Δpsmαβhld*) using an inoculum of 3 × 10^8^ CFUs. Mortality, hematogenous spread (blood culture, spleen and kidney), lung and bone involvements were assessed in two groups (non-survivors of severe sepsis and survivors sacrificed on day (D) 14).

**Results:**

Severe sepsis-related mortality tended to be lower for *Δpsm* derivatives (Kaplan—Meier curves, *P* = .06). Non-survivors’ bone LAC-*Δpsmα* (6.9 log_10_ CFUs/g of bone, *P* = .04) or -*Δpsmαβhld* (6.86 log_10_ CFUs/g of bone, *P* = .014) densities were significantly higher than LAC WT (6.43 log_10_ CFUs/g of bone). Conversely, lung *Δpsmαβhld* CFUs were significantly lower than LAC WT (*P* = .04). LAC *Δpsmα*, *Δpsmαβhld* and WT induced similar bone damage in D14 survivors, with comparable bacterial densities (respectively: 5.89, 5.91, and 6.15 log_10_ CFUs/g of bone). Meanwhile, pulmonary histological scores of inflammation were significantly higher for LAC *Δpsmα*- and *Δpsmαβhld*-infected rabbits compared to LAC WT (*P* = .04 and .01, respectively) but with comparable lung bacterial densities.

**Conclusion:**

Our experimental results showed that deactivating PSM peptides significantly limited bacterial dissemination from bone during the early phase of infection, but did not affect local severity of USA300 rabbit osteomyelitis.

## Introduction

Since 1990, extensive spread in the United States of the community-associated methicillin-resistant *Staphylococcus aureus* (CA-MRSA) USA300 clone has been responsible for severe infections, including bone and joint infections (BJIs), especially in children [1]. BJIs represent up to 38% of pediatric CA-MRSA infections in the United States [2]. The global severity of CA-MRSA BJIs has been linked to local musculoskeletal involvement, including extraosseous abscesses, and the frequent need for surgical management [3,4], and systemic complications including severe sepsis and dissemination to the lungs [5,6].

Numerous CA-MRSA virulence factors have been identified [7], some of which appeared to play a specific role in the course of osteomyelitis. Panton—Valentine leukocidin (PVL), a phage-borne pore-forming toxin highly prevalent in CA-MRSA, was able to induce local complications, e.g. bone deformation and muscular abscesses in a rabbit osteomyelitis model [8]. PVL involvement in this context was further supported by the epidemiological association of PVL expression and clinical methicillin-susceptible *S*. *aureus* BJI severity [1]. CA-MRSA overexpression of another pore-forming toxin, *α*-hemolysin (Hla), was also shown to contribute to BJI pathogenesis [9]. We previously showed that Hla was associated with systemic complications, like severe sepsis-related mortality in CA-MRSA rabbit osteomyelitis [10].

In addition to PVL and Hla, secreted peptides called phenol-soluble modulins (PSMs) have been identified as key virulence factors that are also strongly expressed in staphylococci and CA-MRSA [11]. PSM-encoding genes appear in three distinct loci on the *S*. *aureus* chromosome. PSMs encoded by the first two loci have been designated PSM*α* and *β*. The third PSM locus encodes the *δ*-toxin; its open-reading frame is part of the RNAIII effector of the staphylococcal accessory-gene regulator (*agr*), a major two-component system coupled to a density-sensing cassette controlling the expression of most *S*. *aureus* virulence factors. All PSM-encoding genes are under *agr* control, either through the AgrA-mediated regulation pathway for *psmα* and *β*, or as a consequence of the co-transcription with RNAIII, as for *hld* [12]. Thus, PSM expression is tightly coupled with staphylococcal quorum sensing through *agr*.

Several PSM biological functions impact pathogenesis and, possibly, the course of *S*. *aureus* osteomyelitis. PSMs are small peptides with amphipathic properties, allowing them to destabilize lipid bilayers at high concentrations. This activity has been linked to receptor-independent cytotoxicity to host cells, including neutrophils and osteoblasts, the bone-forming cells [13,14]. Moreover, receptor-dependent proinflammatory activation of neutrophils by PSMs was found, resulting from PSM detection by the neutrophil formyl-peptide receptor 2 (FRP2) [15]. Finally, PSMs can assemble into amyloid-like fibrillae, which contribute to stabilizing staphylococcal biofilms [16,17] and induce a tolerogenic phenotype in dendritic cells, contributing to bacterial interference [12] and cell-cycle disruption [18]. These cytotoxic, proinflammatory and biofilm-enhancing properties could suggest PSM involvement in the *in vivo* outcome of osteomyelitis.

In previous *in vivo* studies on PSMs using a model of skin-and-soft-tissue infection, the LAC *psmα*-deleted strain, but not the other *psm*-deleted strains, was significantly less able to cause skin lesions in mice [11]. Also, Kobayashi et al demonstrated that PSM*α* and Hla contributed to the pathogenesis of USA300 skin infections in rabbits [19], whereas PSM*α* peptides had no impact in a PVL-negative ST72 CA-MRSA strain in a mouse model of skin infection [20]. Moreover, PSMs have been shown to facilitate dissemination from an infected catheter in a mouse model of biofilm-associated infection [21]. Furthermore, in a rabbit model of experimental endocarditis, Spaulding et al showed that deactivating PSMs delayed lethal sepsis but did not prevent mortality or valve lesions [22] and, thus, that they do not play a major role in infective endocarditis.

Concerning BJIs, PSM*α* expression was associated with extensive bone damage inducing the death of infected human osteoblasts in an *ex vivo* model of intracellular infection [14], whereas in a murine osteomyelitis model, PSMs enhanced cortical bone destruction [23]. Addressing *in vivo* PSM BJIs requires using a model sufficiently close to the human situation in terms of PVL susceptibility. In particular, murine models used in previous studies of PSM involvement in CA-MRSA osteomyelitis could not account for the PVL effect, because murine immune cells respond poorly to PVL [24], unlike those of rabbits that are highly PVL-sensitive [25]. Hence, disease outcomes as a function of PVL may differ between these species.

In this context, we sought to determine the contribution of PSMs to local and systemic osteomyelitis severity in a PVL-sensitive rabbit model of acute BJI, using the highly virulent USA300 CA-MRSA strain LAC, which expresses PSMs, Hla and PVL, and its isogenic derivatives lacking PSM expression.

## Materials and Methods

We used the clinical *S*. *aureus* Los Angeles County wild-type strain (LAC-WT), and its isogenic *Δpsm* derivatives (LAC *Δpsmα* and LAC *Δpsmαβhld*, respectively), all kindly provided by Frank Deleo, as for our previous studies. The strains were created and originated in Dr. Michael Otto’s laboratory and were previously described [11].

The *Δpsm* derivatives were constructed via allelic replacement with a spectinomycin-resistance cassette of the *psmα* and *psmβ* operons, as previously described [26], and by disrupting codon usage within the *hld* gene inside RNAIII to disable *δ*-toxin. Technical difficulties and cost prevented us from verifying modifications/confirming plasmid transfer with a gene-recomplementation assay.

Microorganisms were stored at –80°C until use. Prior to the experiments, bacteria were cultured in casein hydrolysate and yeast extract medium (CCY) at 37°C with shaking for 18 h. After centrifugation, the supernatants were passed through 0.22-*μ*m filters, and the pellets were washed and resuspended in phosphate-buffered saline (PBS). All inocula were quantified by optical density (OD), then serial dilutions were plated on tryptic soy agar (bioMérieux, Paris, France).

Norden’s method [27] was used to induce osteomyelitis in female New Zealand white rabbits, weighing between 2 and 3 kg, housed in individual cages with *ad libitum* access to food and water, in compliance with French legislation on animal experimentation and with the approval of the Animal Use Committee of Maisons-Alfort Veterinary School. Rabbits were anesthetized by intramuscular injection of 25 mg/kg each of ketamine (Virbac, Carros, France) and 2% Xylazine (Bayer Santé, Division Santé Animal, Loos, France). An 18-gauge needle was inserted percutaneously through the right tibial metaphysis into the medullary cavity to aspirate 0.4 mL of bone marrow. Infection was induced by direct injection of 0.1 mL of a sclerosing agent (3% sodium-tetradecyl sulfate), followed by 0.2 mL of inoculum (3 × 10^8^ colony-forming units (CFUs)) and 0.1 mL of saline to rinse the syringe. Fentanyl-patch analgesia was given for 7 days following surgery.

Animals were assigned to receive either LAC *Δpsmα* (*n* = 24) or LAC *Δpsmαβhld* (*n* = 24) to evaluate the *in vivo* impact of PSMs. Nineteen LAC-WT—infected rabbits served as controls.

### Macroscopic Appearance and Bacterial Densities in Bone and Lungs

Animals were monitored daily for general and local signs of osteomyelitis (mobility, leg appearance) and were weighed on inoculum and sacrifice days. Moribund animals (immobile, unable to be aroused from a recumbent position and unable to access food or water) were euthanized by rapid intravenous injection of pentobarbital. Otherwise, rabbits were observed until day (D)14 post-infection to assess PSM impact on the osteomyelitis time course.

Before death, venous blood was drawn for culture and serum samples were stored at –20°C for later antibody-profile determination.

On the day of death, lungs, right leg, spleen and kidneys were removed. Both lungs were weighed and visually examined. Left lungs were stored at –80°C until determination of bacterial densities and right lungs were embedded in paraffin for histological examination (scored 0–4: none, minimal, mild, moderate, severe, respectively) for diffusion, edema, congestion, hemorrhages, thrombi, inflammation, megakaryocytes, infarcts, abscesses, pleural involvement and bacterial density. Spleen and kidneys were crushed and cultured on blood agar to determine their infection status. Macroscopic appearance of the right leg was noted and photographed, and the upper third of the tibia was frozen for subsequent quantitative culture, as previously described [10].

### Serum-Antibody Assay

Anti-PVL and -Hla antibodies were detected with specific enzyme-linked immunosorbent assays (ELISAs). Antibody levels are expressed as arbitrary units per mL (AU/mL), as previously described [10]. Anti-PSM antibodies could not be quantified.

### Statistical Analyses

Percentages of hematogenous spread (positive blood, kidney and/or spleen cultures), bone deformations and abscesses were compared using Student’s *t*-test. Non-parametric Mann—Whitney *U*-tests were used to compare tibial bacterial counts and 2-way ANOVA with Tukey’s multiple comparisons tests for histological scores.

**Fig 1:**
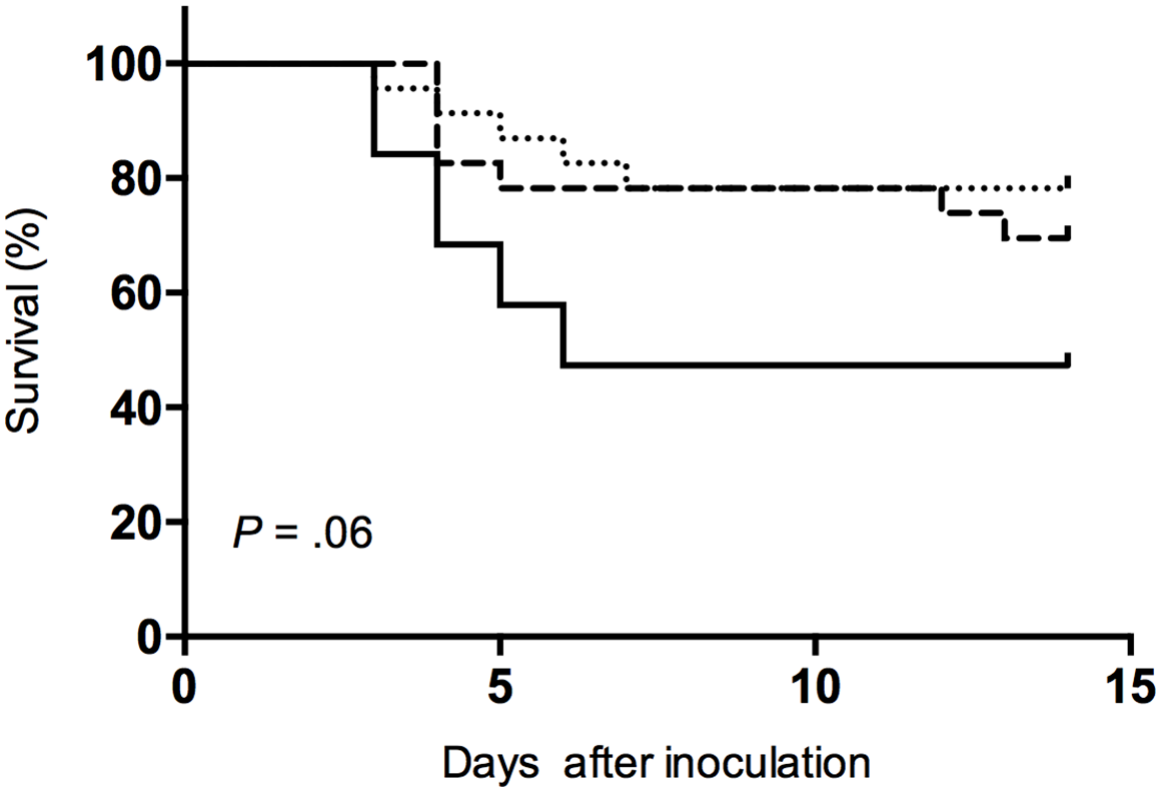
Kaplan—Meier survival curves compared with the log-rank (Mantel—Cox) test. Severe sepsis-related mortality tended to be lower for *Δpsmα* (**··**) and Δ*psmαβhld* (- -) (*P* = .06) vs. LAC WT (–).

The Kaplan—Meier method was used to estimate survival mortality, with inoculation as time 0 and censoring at sacrifice on D14. Percentages of survivors were compared with the log-rank (Mantel—Cox) test (Fig 1).

Survivors’ antibody-titer changes between D0 and D14 were analyzed with paired Welch’s *t*-test after log_10_ transformation.

## Results

We inoculated 48 rabbits with 3 × 10^8^ *Δpsmα* (*n* = 24) or *Δpsmαβhld* (*n* = 24) CFUs; the two that died immediately post-anesthesia (1 each in *Δpsmα* or *Δpsmαβhld* group) were excluded. Nineteen LAC-WT—inoculated rabbits served as controls.

D1 blood cultures were positive for 50% of the LAC-WT—infected rabbits tested vs. 65% of *Δpsmα*-infected (*P* = .47) and 61% of *Δpsmαβhld*-infected animals (*P* = .72).

Ten (52%) LAC-WT—infected rabbits died of severe sepsis with disseminated infection between D0 and D7 (median: 4 days) compared to five (22%) of the *Δpsmα* and seven (30%) of *Δpsmαβhld* groups (*P* = .03 and .11, respectively), with respective median survival of 5 and 4 days (*P* = .53, non-significant (NS)). The lack of significantly different mortality rates between *Δpsmαβhld* and WT groups, as opposed to the significantly different *Δpsmα* group, was likely attributable to random fluctuation and small sample size rather than to a real difference between *Δpsmα* and *Δpsmαβhld* groups. Indeed, Kaplan—Meier survival curves (Fig 1) showed a trend towards lower mortality of *Δpsmα*- and *Δpsmαβhld*-infected rabbits (*P* = .06).

Most non-survivors had positive spleen (80% of *Δpsmα* vs. 100% of *Δpsmαβhld* and LAC WT, NS) and kidney (100% of all groups) cultures. Macroscopic examination of all non-survivors’ lungs found red and congestive lesions, as in the LAC-WT group. Their lung histological scores confirmed the comparability of *Δpsmα* and *Δpsmαβhld* vs. LAC-WT infections. However, LAC-WT bacterial lung densities were higher (8.19 (interquartile range (IQR), 7.55–8.23) log_10_ CFUs/g than *Δpsmαβhld* (6.39 (IQR, 6.27–6.48) log_10_ CFUs/g *P* = .04), but comparable to *Δpsmα* (6.79 (IQR 5.62–7.10) log_10_ CFUs/g, *P* = .25).

**Fig 2:**
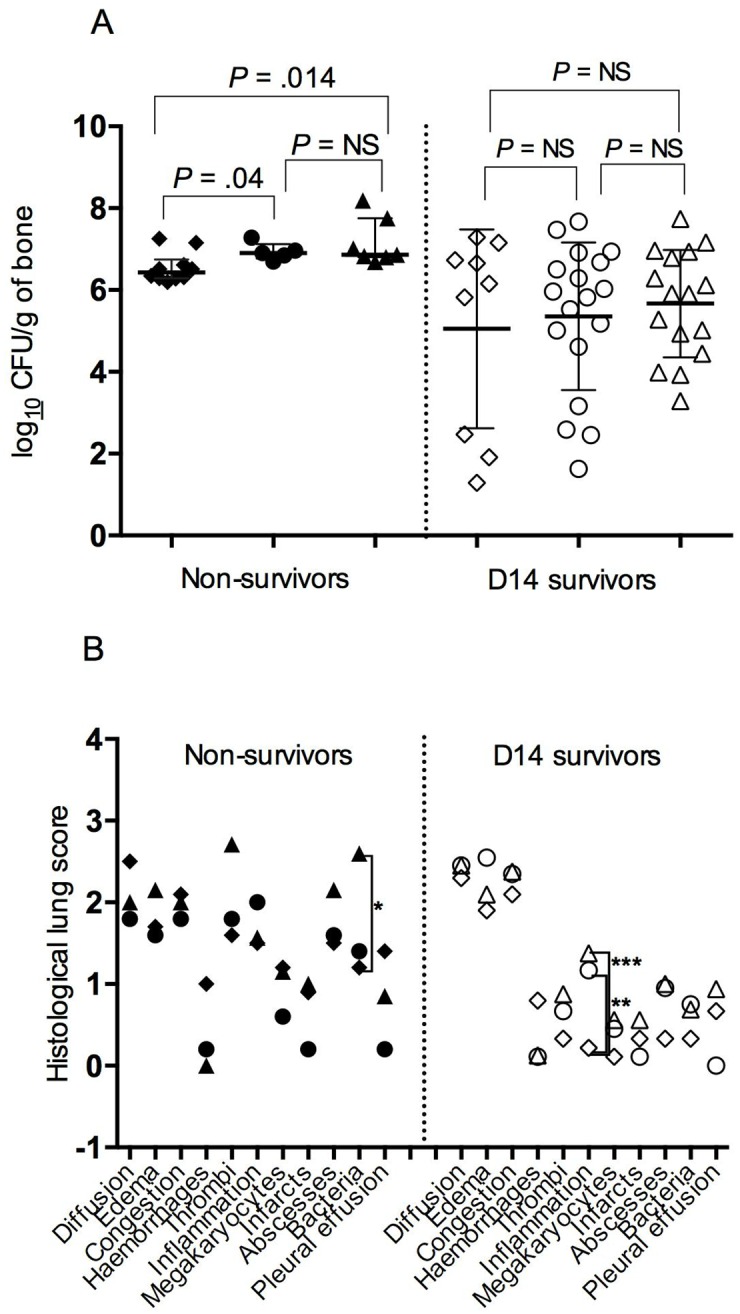
Comparisons of osteomyelitis parameters observed in *Δpsmα* (●○)-, *Δpsmαβhld* ▲△)- and with LAC-WT (◆◇)–infected non-survivors (black) and D14 survivors (white). (**A**) Median bacterial bone densities, expressed in log_10_ CFUs/g of bone. The *Δpsmα*- or *Δpsmαβhld*-infected non-survivors differed significantly from those infected with LAC WT. (**B**) Mean histological scores (0–4: none, minimal, mild, moderate, severe) for lung involvement. The bacterial densities in *Δpsmαβhld*-infected non-survivors differed significantly from those infected with LAC WT (*P = .017), whereas inflammation was more severe in *Δpsmαβhld*- and *Δpsmα*-infected D14 survivors than those infected with LAC WT (**P = .04 and ***.01, respectively).

All non-survivors had infected bones, with median bacterial densities of 6.9 (IQR 6.85–6.96) log_10_ *Δpsmα* CFUs/g and 6.86 (IQR, 6.81–7.38) *Δpsmαβhld* log_10_ CFUs/g vs. 6.43 (IQR, 6.29–6.58) log_10_ LAC WT CFUs/g of bone (*P* = .04 and .014, respectively) (Fig 2A). As for LAC-WT—infected rabbits, rare microabscesses were found at the inoculation site (0% *Δpsmα*, 20% *Δpsmαβhld* vs. 20% LAC WT, NS) but no cortical deformation (NS).

**Fig 3:**
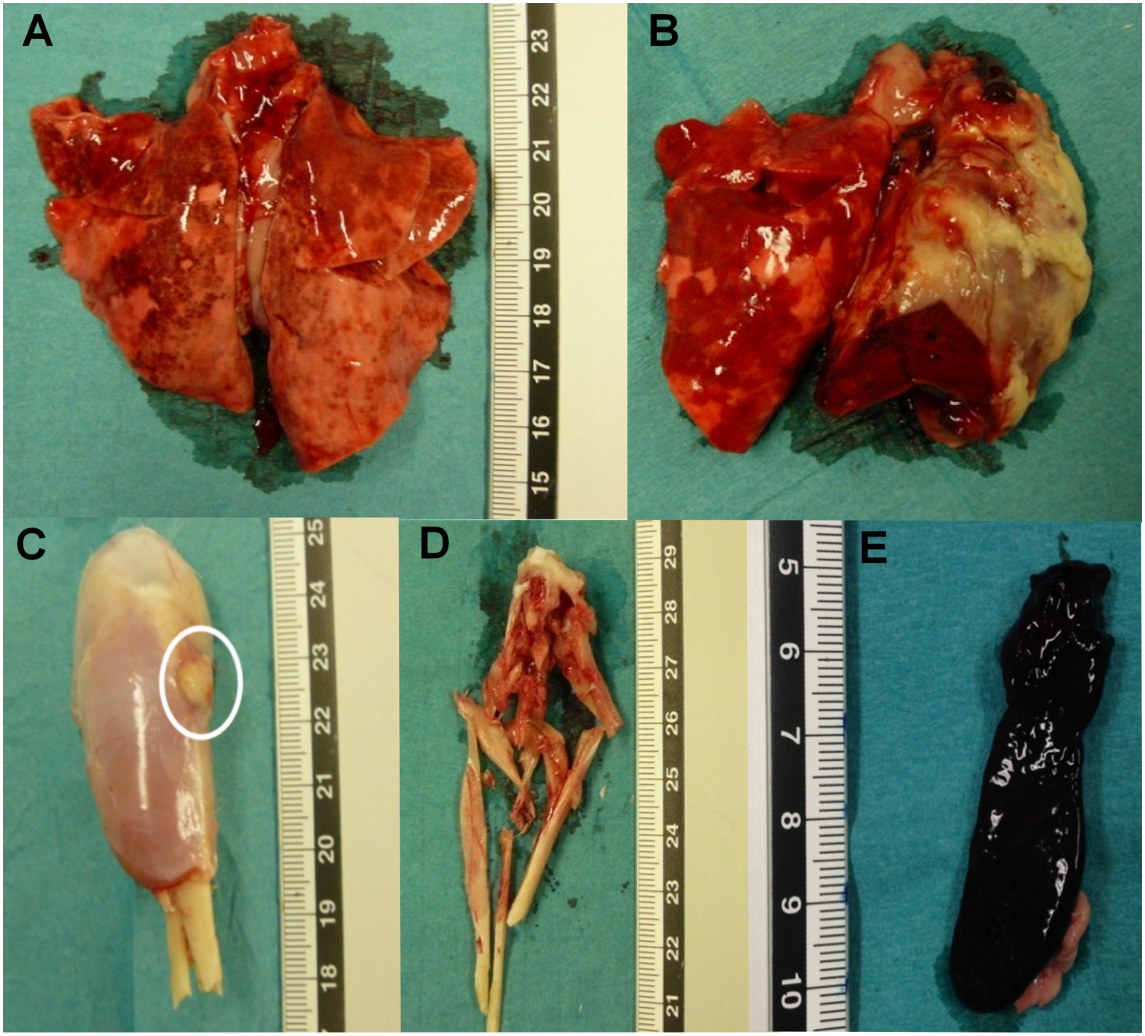
Macroscopic findings after challenge with a high inoculum of CA-MRSA USA300. (**A**) Pulmonary hemorrhages demarcated by hyperemic regions in both lungs. (**B**) Abscesses in the left lung indicating disseminated infection. (**C**) White circled muscle abscess in the right leg. (**D**) Bone marrow filled with pus indicating osteomyelitis. (**E**) Splenomegaly with necrosis observed after disseminated sepsis.

Survivors were sacrificed on D14 to evaluate bones and organs (Fig 3).

D14 *Δpsmα*- and *Δpsmαβhld*-infected survivors did not differ from LAC-WT—inoculated rabbits for hematogenous dissemination: 17%, 19% vs. 11% spleen-positive cultures (NS) and 34%, 44% vs. 11% positive kidney cultures (NS). Histological scores were comparable except for higher lung inflammation in *Δpsmα* (*P* = .04) and *Δpsmαβhld* (*P* = .01) than LAC-WT infections (Fig 2B), while mean (±SD) lung bacterial densities were comparable for the three groups (2.31 ± 1.04, 2.21 ± 0.39 vs. 2.03 ± 1.18 log_10_ CFUs/g, respectively; NS).

Unexpectedly, *Δpsmα* and *Δpsmαβhld* vs. LAC-WT strains, respectively, induced similar bone deformities (39% and 43% vs. 67%; *P* = .73 and .41), muscle abscesses (83% and 75% vs. 89%; NS), and bone bacterial density (5.89 (IQR, 4.71–6.63) log_10_ CFUs/g and 5.91 (IQR, 4.82–6.83) log_10_ CFUs/g) vs. (6.15 (IQR, 2.47–6.73) log_10_ CFUs/g) (*P* = .95 and .82) (Fig 2A).

**Fig 4:**
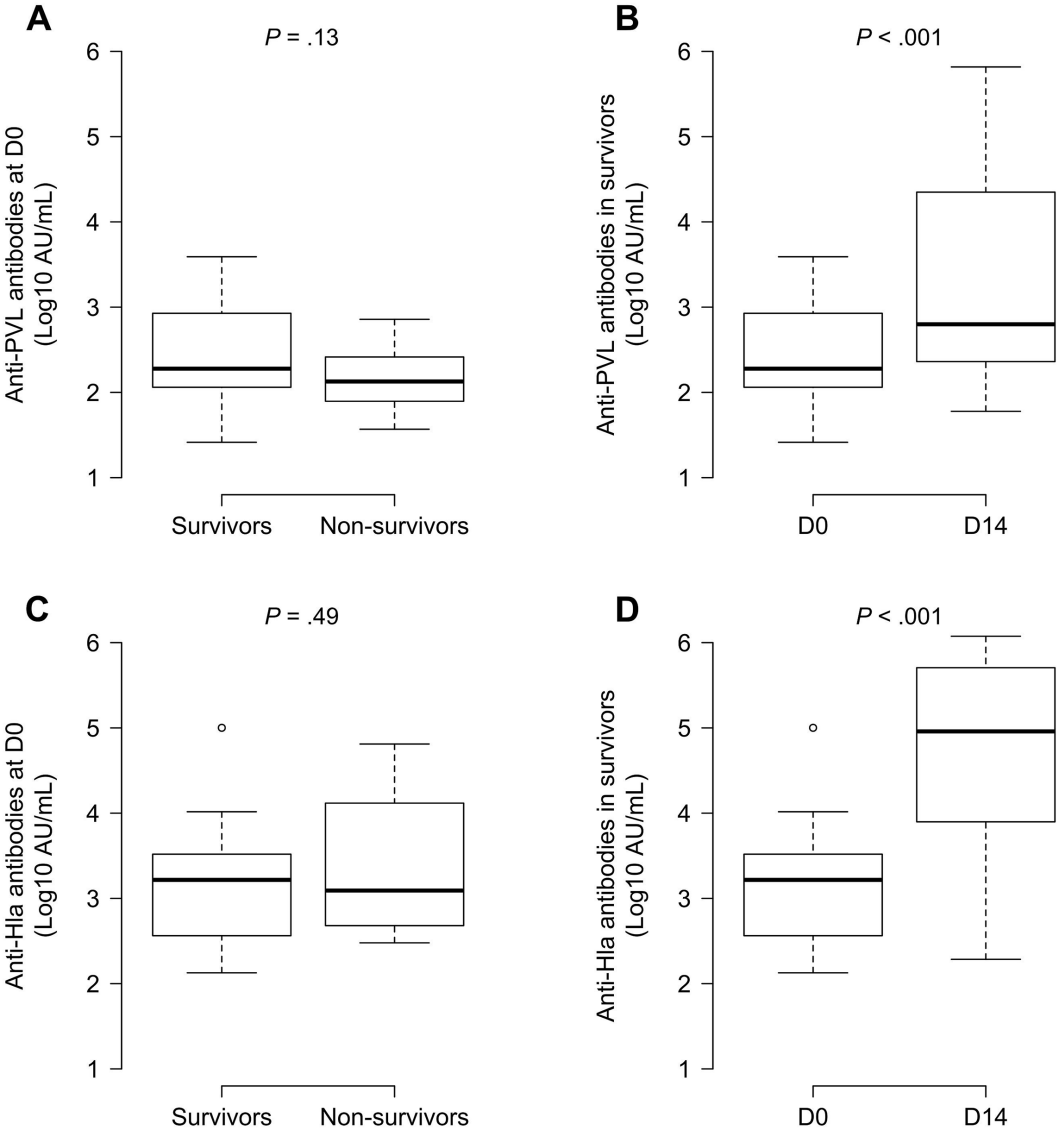
Serum anti-PVL—and -Hla—antibody titers in a rabbit model of *S*. *aureus* LAC experimental osteomyelitis. Survivors’ and non-survivors’ Initial anti-PVL (**A**) and -Hla titers (**C**) did not differ significantly. Survivors had significantly higher anti-PVL (**B**) and -Hla (**D**) antibody levels between D0 (inoculation) and D14 (sacrifice) (*P* < .001). Welch’s *t*-test analyses of paired (**B**, **D**) or unpaired (**A**, **C**) log_10_ values.

D14 survivor’s titers were significantly higher than on D0 for anti-PVL (6.4-fold increase, 95% CI 2.9, 14.1; *P* = 3.5 × 10^−5^, paired *t*-test of log_10_ values) and anti-Hla antibodies (41.3-fold increase, 95% CI 18.1, 96.8; *P* = 2.9 × 10^−10^), thereby confirming a significant immune response towards the two toxins. Survivors’ and non-survivors’ D0 anti-PVL and anti-Hla antibody titers were similar (Fig 4).

## Discussion

Our experimental model closely reproduces features of severe acute osteomyelitis seen in children with about half of the LAC-WT—infected rabbits dying within 7 days of severe sepsis, bacteremia, and high bone and lung bacterial densities associated with histological lung lesions, while D14 survivors developed severe bone infections with deformation and abscesses.

Our main results demonstrated that, during the early first phase of acute disseminated severe sepsis, deactivating PSMs tended to limit sepsis-attributable mortality, albeit not significantly. *Δpsmαβhld*-infected non-survivors had more bone CFUs than LAC-WT—infected non-survivors. In contrast, the former non-survivors had lower lung CFUs. This result can appear to be inconsistent with the bacterial lung histological score. However, bacterial enumeration by CFU counting is more reliable because (i) based on a larger sample size (tissue homogenate of a large piece of tissue) and (ii) assessing living but not dead cells.

These findings agree with the previously described role of PSMs in facilitating bacterial dissemination from an infected catheter in a mouse model [21, 28], a biofilm-associated infection like BJI. Conversely, during the second phase of subacute osteomyelitis (D14), deactivating PSMs did not modify bone CFUs, abscesses or deformities, in contrast to what could have been anticipated based on previous *in vitro* and *ex vivo* observations of PSMs’ osteoblast cytotoxicities [14].

The impact of PSMs (mainly PSM*α*) during the early phase of severe sepsis could be due to elevated production of *agr*-dependent toxins, including Hla and PSMs [29], especially by the LAC-WT strain. Although PSM deletion resulted in lower mortality, the effect was not significant, in contrast to previous observations with Hla [10].

Alternatively, the absence of PSMs’ effect on bone damage in D14 survivors could be explained by two hypotheses. First, *agr* might be less expressed during this late phase of osteomyelitis, considered a localized biofilm infection [29]. That possibility underlines the need to study the impact of virulence factors in a subacute osteomyelitis model (D14) with long-term animal survival [14]. Second, a potential PSM effect could have been masked by the impact of PVL, which plays a major role by enhancing rapid local spread of rabbit osteomyelitis with extraosseous infection extension, especially muscle abscesses [8], or by other toxins, e.g. Hla, which has been associated with severe sepsis-related mortality [10]. PSMs are by far the most abundant protein secreted by *S*. *aureus* (70–80%), much more than Hla or PVL [30]. Our results do not exclude a possible PSM effect on osteomyelitis caused by PVL-negative *S*. *aureus* isolates. A double-mutant strain *Δpvl–Δpsmαβhld* would have helped test this hypothesis, but we were unsuccessful in constructing one.

Our results do not corroborate those of Cassat et al, who showed that PSM*α* significantly limited bone remodeling in mice after creation of a cortical defect in the femur and local inoculation of 1 × 10^6^ LAC-WT or *Δpsmα1–4* CFUs [23]. However, it should be stressed that their model of post-traumatic localized osteomyelitis is far from what was encountered in children with primary CA-MRSA osteomyelitis [1,2,4,5,31].

Finally, because PSMs were shown to play a proinflammatory role by inducing neutrophil activation and cytokine release through the through human formyl-peptide receptor-2 (FPR2) pathway, the higher pulmonary inflammation scores for *Δpsmα* and *Δpsmαβhld* groups were also unexpected [15]. Our results cannot be explained by the comparable lung bacterial densities of *Δpsmαβhld* and LAC-WT groups. FPR2 is known to be expressed by several epithelial tissues including human, mouse and rat lungs. However, FPR expressed by rabbits is only 68% homologous to FPR2. Also, FPR2 can be triggered by different ligands and induce pro- or anti-inflammatory responses [32]. Therefore, we think the pulmonary inflammation seen in experimental rabbit osteomyelitis might involve other pathways and cytokines, which would support our results.

In conclusion, our results showed that deactivating PSMs prevented bacterial dissemination from bone during the early stage of the infection, but did not impact the local severity of rabbit USA300 osteomyelitis during the later stage. In agreement with our earlier work on PVL and Hla involvements in CA-MRSA osteomyelitis [8,10], these new findings confirmed that PVL expression in a susceptible host, e.g. rabbit, is the major cause of abscesses and bone damage, potentially masking PSM effects. Nevertheless, PSMs and their modulation by the staphylococcal PSM-degrading protease aureolysin might remain important bone-damaging factors in PVL-insensitive hosts [23] and perhaps in PVL-negative *S*. *aureus*. This strong dependence of staphylococcal osteomyelitis pathophysiology on host susceptibility factors and the infecting strain’s toxin-gene content are exemplary of the difficulties that must be overcome to improve our understanding of this disease. Beyond the pathogenic role of toxins, other bacterial factors related to quorum sensing and hypoxic response in poorly oxygenated bone tissue are likely equally important to establish bone infection, as recently demonstrated by comparisons of *S*. *aureus* genes essential to infection in abscess and osteomyelitis models [33,34]. Among the intricate array of staphylococcal virulence factors known to contribute to osteomyelitis so far, Hla is the only one that: measurably influences local and systemic outcomes, even in the presence of PVL [10]; is common to virtually all *S*. *aureus* lineages [35]; and is already targeted by a monoclonal antibody being tested in a clinical trial (study identifier NCT02296320) to treat *S*. *aureus* infection [36]. In this context, it seems reasonable to designate Hla as a more promising target than PSMs to treat severe CA-MRSA osteomyelitis, in agreement with recent conclusions drawn from a mice model of skin and soft tissue infection [20].
